# Evaluation of Protein Profiling in a Cohort of Egyptian Population with Renal Cell Carcinoma and Benign Kidney Neoplasms

**DOI:** 10.31557/APJCP.2019.20.7.2145

**Published:** 2019

**Authors:** Noha Said Kandil, Rasha Abdelmawla Ghazala, Rania Mohamed El Sharkawy, Tamer Abou Youssif, Noha Abouseda

**Affiliations:** 1 *Department of Chemical Pathology, Medical Research Institute, *; 2 *Department of Biochemistry, *; 3 *Department of Urology, * ^*4*^ *Department of Microbiology, *; 4 *Department of Microbiology, Faculty of Medicine, Alexandria University, Egypt. *

**Keywords:** Magnetic Beads- MALDI TOF- proteomic profiling- renal cell carcinoma

## Abstract

**Section Title:**

Abdominal imaging leads to the detection of a large number of renal tumors without the ability to distinguish the type of tumor detected. It is necessary to find a precise way to know the type of tumor to determine the appropriate treatment. The use of urine samples for detecting new biomarkers especially proteins has a great potential. In this work we assessed the proteomic profiling difference in a cohort of Egyptian population with renal neoplasms.

**Methods:**

This cohort study was conducted on 85 subjects. They were classified as 40 RCC, 15 benign kidney patients, and 30 healthy controls. Morning urine samples were used for peptidome separation using magnetic beads. Matrix assisted laser desorption ionization time of flight mass spectrometry (MALDI-TOF MS) was applied Using FlexControlTM software.

**Results:**

Benign tumors were differentiated from controls by 5 integrated peaks, 12 significant and 2 integrated significant peaks, 17:3,418.8 and 25:4,173.41. While RCC were differentiated from benign by 5 integrated, 28 significant and one integrated significant peak. The RCC group was discriminated from the controls by 5 peaks which were integrated from which 1 was integrated and significant (with mass to charge ratio of 12:3,408.97). The three groups showed protein profiles ranging from 1 to 10 kDa. The external validation was performed for the RCC group versus the control reveled sensitivity of 88.7% and specificity of 73.2% by genetic algorithm.

**Conclusion:**

Proteomic approach can be used as a sensitive urinary marker differentiating renal masses in an early diagnostic approach.

## Introduction

Kidney cancer is considered the seventh most common cancer worldwide representing 3.3% of all types of cancer. Renal cell carcinoma (RCC) accounts for more than 90% of all kidney neoplasms (Wong et al., 2017). There is wide variability in the incidence rate of RCC, being highest in North America and least in Africa (Jonasch et al., 2014). It has high morbidity and mortality with a five year survival time not exceeding 10% (Hosoya et al., 2013). Moreover more than one third of the cases are diagnosed in late stages of the disease after tumor metastasizing, also it has a high recurrence rate exceeding 30% of the cases with localized kidney cancer (Chinello et al., 2015). Several risk factors are participating in the development of RCC including age, sex, socioeconomic status, genetic predisposition, cigarette smoking, obesity, hypertension and alcohol intake (Kabaria et al., 2016). 

The incidental discovery of RCC accounts of more than 50% of the diagnosed cases. This is attributed to the extensive utilization of various radiological techniques such as abdominal ultrasound (U.S), computed tomography (C.T) and magnetic resonance imaging (M.R.I) (Escudier et al., 2019).

The role of the laboratory was confined to cases presenting with suspicious symptoms of RCC known as the classical triad, including gross hematuria, flank pain and palpable abdominal mass with no role in early diagnosis. These tests include; urine analysis, serum creatinine, hemoglobin, leukocyte and platelet counts, lactate dehydrogenase and serum corrected calcium, together with C-reactive protein (CRP) and erythrocyte sedimentation rate. These tests together with Tumor-Node-Metastasis staging (TNM) after surgical removal of the tumor were used to assess the prognosis, recurrence, survival rate and risk assessment (Escudier et al., 2019; Karam et al., 2011). 

The use of noninvasive biological sample that helps in early diagnosis of urological malignancies is becoming a necessity. The use of urine samples for detecting new biomarkers, has been widely used owing to its unique nature, being accessible with abundant volumes in an easy noninvasive technique (Decramer et al., 2008; Beasley-Green, 2016). Proteins are normally excreted in urine at concentrations less than 150 mg/L per 24. Two third of these proteins come from the kidney and one third from ultrafiltration of the plasma (Thongboonkerd and Malasit, 2005). 

Urinary proteomic assessment is very important as it helps in identifying the nature of renal and non-renal diseases. One of the main constrains regarding the use of urinary proteome as a biomarker, is the presence of large number of proteins in a normal urine sample. Moreover many factors influence identification of protein/peptide biomarkers in urine such as sex, age, diet, lifestyle, and physiological condition (Beasley-Green, 2016).

All these challenges led the scientists to explore the diverse mass spectrometric (M.S) techniques to characterize urine proteome. These techniques differ from one another in many methodological performance degrees concerning sensitivity, specificity, reproducibility and mass resolution (Beasley-Green et al., 2012). Many M.S techniques have been used in the assessment of urinary proteome including both the targeted and untargeted approach. Among these techniques the mostly used ones are, the matrix-assisted laser desorption/ionization-time-of-flight (MALDI-TOF) MS, high-resolution MS and triple quadrupole MS (Beasley-Green, 2016; Beasley-Green et al., 2012).

MALDI-TOF application in urine proteome has many advantages as it can be used in targeted and global profiling also it is considered a perfect approach when using simple matrix but on the other hand testing the urine samples demands considerable sample preparation as desalting, enrichment, fractionation, and separation. These preparations are required to reduce the complexity of the proteome, avoid under sampling and suppression of low abundant proteins (Pieper et al., 2004). 

This Research aims at assessing the proteomics profiling difference in a cohort of Egyptian population with renal neoplasms. 

## Materials and Methods

This study was conducted on 85 subjects, aged between 41-73 years (mean of 54 years ), admitted to urology department, faculty of Medicine. They were classified as 30 healthy controls, 40 clear cell RCC patients (ccRCC) and 15 benign kidney diseases. Renal, urinary tract infection and any other renal diseases were excluded .After approval of the ethical committees of the Medical Research Institute and the Faculty of Medicine, Alexandria University; the study was conducted in accordance with the Declaration of Helsinki and the International Conference on Harmonization Guidelines for Good Clinical Practice. Informed consent was obtained from all eligible subjects before starting the study.

All subjects had signed an informed consent prior to sample donation. Patients were diagnosed as RCC based on radiological findings of C.T and classified according to the 2009 TNM system classification and their clinical characteristics. Only cases with T1 and T2 with masses not exceeding 7 cm were the present study. As for the benign renal adenoma it was diagnosed histopathologically.

*Exclusion criteria*

Chronic renal failure 

Other malignancies

History of malignancy

Abnormal urine analysis.

*Reagents*

Magnetic bead hydrophobic-interaction chromatography (MB-HIC8) beads, that separate low molecular weight peptides according to the size, were purchased from Bruker daltonics.

Gradient-grade acetonitrile and ethanol, and pro analysis–grade trifluoroacetic acid, urea, sodium chloride, and acetone were used from Sigma-Aldrich. Clinprot standard CPS (Peptide Calibration Standard II and Protein Calibration Standard I) and cyano-4-hdroxycinnamic acid (HCCA)(Bruker Daltonics) were used in profiling experiment. 

*Urine collection and storage*

Morning urine samples were collected in 100-mL urine cups .After sample collection the cup was placed in an ice pack for transport. The sample was then centrifuged in a cooling centrifuge at 5°C for 15 minutes at 1,800 xg. Then, samples were aliquoted and stored immediately at -80°C till further analysis after determination of their total protein content. 

*Peptidome separation*

Urine samples were thawed at room temperature for30 min, centrifuged again. The total protein in urine samples were adjusted to 3.5 µg by varying sample volume to reach 30 µl by the Binding Buffer (BB). MB–HIC C8 purifications were performed according to the manufacturer’s protocol for urine peptidome separation. The peptide fraction was eluted from beads with 5 µL acetonitrile/water 1:1.

*MALDI-TOF MS Experiment*

MALDI-Matrix α-cyano-4-hydroxycinnamic acid (HCCA) was chosen for peptide profiling experiment on polished steel targets. 1 µl of sample was applied to a target spot and left to dry at room temperature Then, 1 µl of matrix was applied on the spot. The mixture was then left to dry at room temperature. Spectra acquisition was done using the positive linear mode (1-10) kDa of the MALDI-TOF/TOF UtrafleXtreme mass spectrometer from Bruker Daltonics (Bremen, Germany).For optimum performance, the ClinProt standard (CPS) was used as a standard sample. Using the FlexControlTM software, peaks with signal/noise ratio (S/N) ratio above 3 were only chosen from the spectra generated.

*Expression profile analysis and statistical analysis*

The ClinPro Tools software 3.0 (Bruker, Daltonik, Germany) was used for analysis and data processing. The mean spectrum obtained from each subject data set was used for the statistical elaboration. The calculated p-values, less than 0.05, were considered to be significant. Then a class prediction model was set up by genetic algorithm (GA), supervised neural network (SNN) and quick classifier (QC) algorithms. Cross -validation were implemented to determine the accuracy of the class prediction. 

## Results

The urinary peptidome profile for 40 renal cell carcinoma patients , 15 benign kidney neoplasm and 30 healthy controls were analyzed in this research .This study highlighted the difference in profiling between the patients either malignant or benign versus the healthy control in a trial of detection of early diagnostic biomarker for RCC or benign kidney disease. ClinProtTools version 3.0 was used for the proteomic analysis.

**Table 1:** Representing the GA , SNN and QC Models for the Studied Groups and the Validation Set

Name	cross validation	sensitivity	specificity	Recognition Capability
Benign versus the control				
SNN	82%	79.50%	84.60%	98.7
GA	98.60%	97.30%	100%	100
QC	92.50%	98.60%	86.30%	91.70%
RCC versus the benign				
SNN	50%	0%	100%	50.40%
GA	85.90%	96%	75.90%	99.60%
QC	86.80%	74.80%	98.80%	85.10%
RCC versus the control				
SNN	50%	0%	100%	50.40%
GA	97.50%	100%	95%	100%
QC	86.80%	74.80%	98.80%	85.10%
Validation study				
GA	Sensitivity	88.70%	Specificity	73.20%

**Table 2:** Comparison between the benign versus the Control Groups for Proteomic Analysis

	Total number of peaks	No/ number : masses of integrating regions using the GA model		No of significant peaks (PWKW < .05)	No of significant integrated peaks	Sensitivity	specificity
Benign versus the controls	64	5 peaks	8:1,913.17,17:3,418.8, 15:3388.67,4:1525.1,25:4173.41	12 peaks	17:3,418.8,25:4173.41	97.26%	100%

**Table 3:** Comparison between the RCC versus the benign Groups for Proteomic Analysis

	Total number of peaks	No/ number : masses of integrating regions using the GA model		No of significant peaks (PWKW < 0.05)	No of significant integrated peaks	Sensitivity	Specificity
RCC versus the benign	65	5 peaks	7:1682, 40:5247.36, 27:4309.24, 18:3428.41 , 24:4101.63	28 peaks	40:5247.36,27:4309.24	95.96%	78.86%

**Table 4:** Comparison between the RCC versus the Control Groups for Proteomic Analysis

	Total number of peaks	No/ number : masses of integrating regions using the GA model		No of significant peaks (PWKW < 0.05)	No of significant integrated peaks	Sensitivity	Specificity
RCC versus the controls	60	5 peaks	21:4173.37, 35:5246.64 , 12:3408.97 , 24:4308.97 , 2:1682.04	5 peaks	08:49.0	100%	95.40%

**Figure 1-A:**
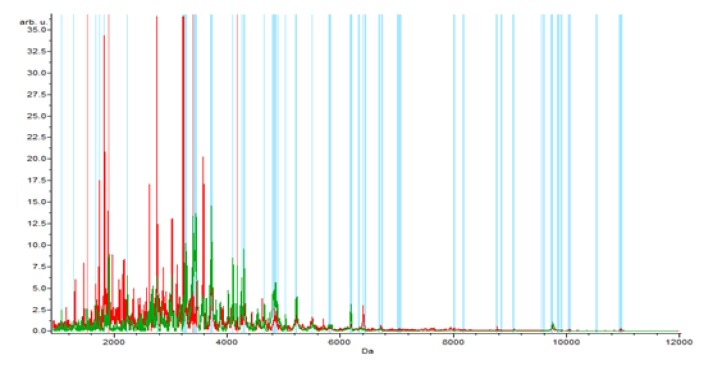
The Whole Spectral View Using C8 in ClinPro Tools. The figure represents class I (benign) in red against class II (control) in green. Included peaks are demarcated with vertical blue lines. Peaks demarcated with red vertical lines are the integration regions

**Figure 1-B:**
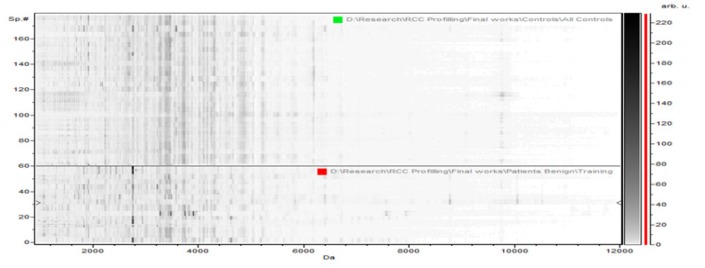
The Pseudogel View Using C8 in ClinPro Tools. The figure shows class I (benign) at the bottom and class II (controls) in the top. Each peak is represented by a vertical line. The difference in intensity between the lines in class I and class II represents the differential peak expression between the two classes

**Figure 1-C:**
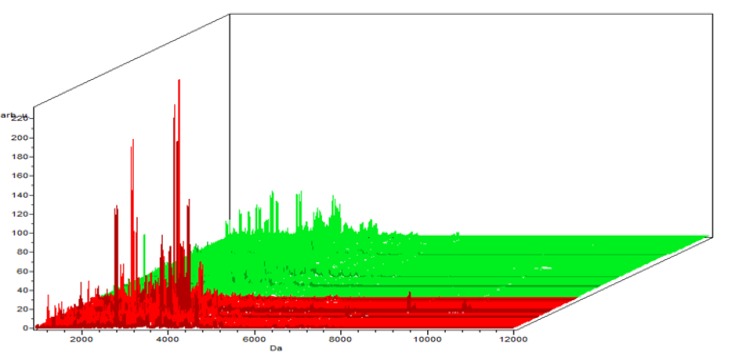
The Colored Stack View Using C8 in ClinPro Tools. It shows the spectra of class I (benign ) in red and Class II (controls)

**Figure 2-A:**
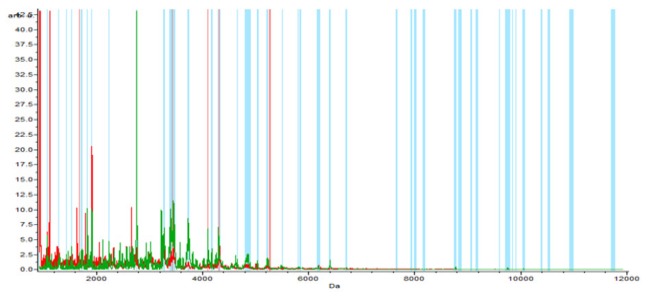
The Whole Spectral View Using C8 in ClinPro Tools. The figure represents class I (RCC) in red against class II (benign) in green. Included peaks are demarcated with vertical blue lines. Peaks demarcated with red vertical lines are the integration regions

**Figure 2-B:**
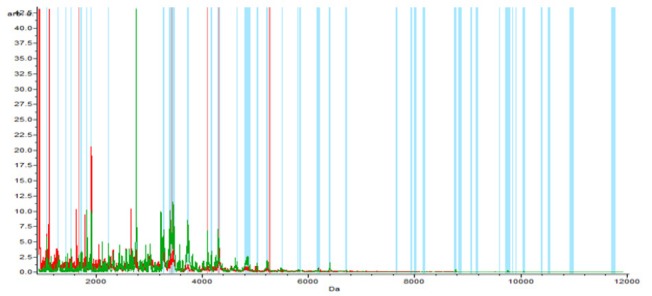
The Pseudogel View Using C8 in ClinPro Tools. The figure shows class I (benign) at the bottom and class II (controls) in the top. Each peak is represented by a vertical line. The difference in intensity between the lines in class I and class II represents the differential peak expression between the two classes

**Figure 2-C:**
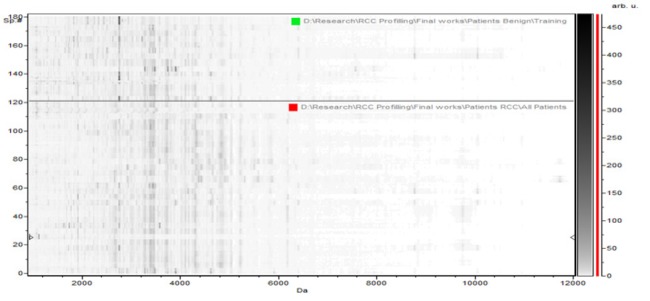
The Colored Stack View Using C8 in ClinPro Tools. It shows the spectra of class I (RCC ) in red and Class II (benign)

**Figure 3-A:**
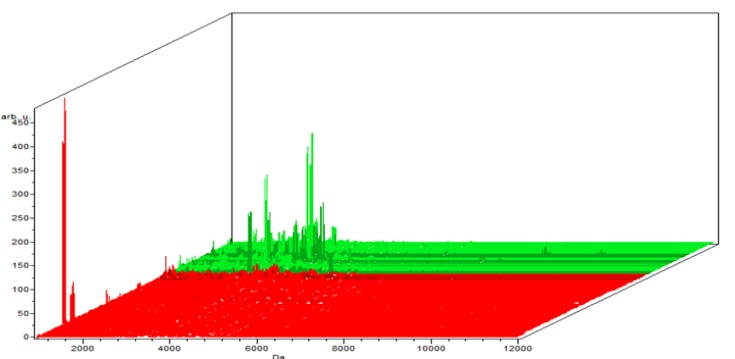
The Whole Spectral View Using C8 in ClinPro Tools. The figure represents class I (RCC) in red against class II (control) in green. Included peaks are demarcated with vertical blue lines. Peaks demarcated with red vertical lines are the integration regions.

**Figure 3-B:**
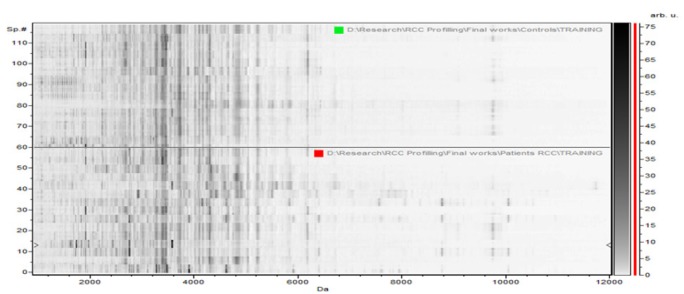
The Pseudogel View Using C8 in ClinPro Tools. The figure shows class I (RCC) at the bottom and class II (controls) in the top. Each peak is represented by a vertical line. The difference in intensity between the lines in class I and class II represents the differential peak expression between the two classes.

**Figure 3 -C:**
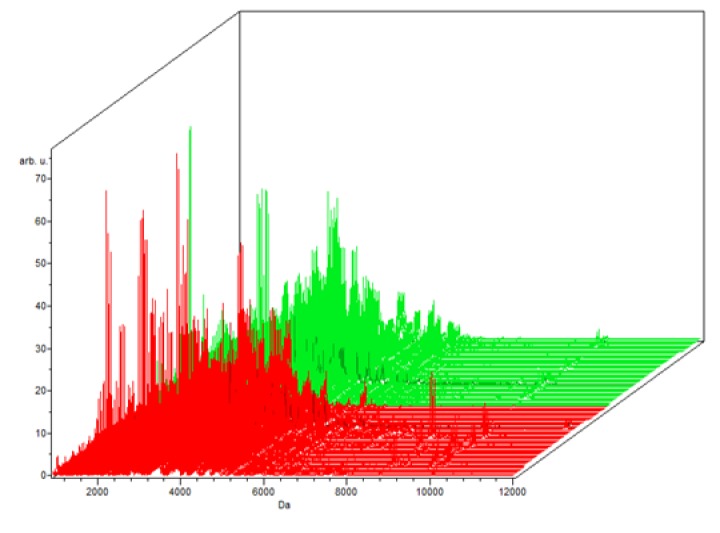
The Colored Stack View Using C8 in ClinPro Tools. It shows the spectra of class I (RCC ) in red and Class II (control)

*MALDI spectrum generation*

Sample separation by C8 followed by fractionation by MALDI-TOF MS identified 64, 65 and 60 peaks respectively. Using the spectral data from the three groups, three different classification models for the three groups were generated using GA, SNN and QC algorithms. The GA model showed the best sensitivity and specificity in the three trials (Table 1). 

The Benign versus the control data, the results revealed 64 peaks that discriminated the benign from the control group; 45 upregulated and 19 peak down regulated. Integration regions used for classification were the following peaks with mass to charge ratio of (8:1,913.17, 17:3,418.8, 15:3,388.67, 4:1,525.1, 25:4,173.41), 2 were integrated and significant (PWKW p> .0. 0001) which are17:3,418.8 and 25:4,173.41. Peaks of m/w 8:1,913.17 and 4:1,525.1 were upregulated and 17 :3,418.8, 15:3,388.67, 25: 4,173.41 were downregulated (Table 2).

Regarding the RCC versus benign; 65 peaks were identified. 50 upregulated and 15 downregulated peaks. Integration regions used for classification were the following peaks with mass to charge ratio of (7:1682, 40:5,247.36, 27:4,309.24, 18:3,428.41, 24:4,101.63), one only was significant PWKW (p> .0. 0001). All the integrated peaks were downregulated except peak 18:3428.41 was upregulated (Table 3).

Finally, the RCC group was discriminated from the controls group, the trial showed 60 peaks. 36 were upregulated and 24 were downregulated. Five were chosen (with mass to charge ratio of 21:4,173.37, 35:5,246.64, 12:3,408.97, 24:4,308.97, 2:1,682.04), from which 1 was integrated and significant (with mass to charge ratio of 12:3,408.97) (PWKW p> .0. 0001)), from which peak 35:5246.64 and 24:4308.97 were up regulated on the other hand 21:4,173.37 ,12:3,408.97 and 2:1,682.04 were down regulated (Table 4).

In the component analysis, the spectra view, pseudo gel and the stack view were done for the three trials benign versus controls , RCC versus benign and RCC versus control as shown in the Figures (1, 2, 3) respectively. The external validation was performed for the RCC group versus the control reveled sensitivity of 88.7% and specificity of 73.2%

## Discussion

Renal cell carcinoma is the seventh most common cancer in the world (Jemal et al., 2011; Wong et al., 2017). More than one quarter of the cases are first presented with metastatic disease, owing to this it was mandatory to have a more sensitive method to detect RCC at early stage before metastasis (Wong et al., 2017). Most cases are frequently detected as an incidental renal mass during abdominal imaging for other reasons. There is clearly a need for clinically useful biomarkers to allow earlier detection, stratification /prioritization of investigation in symptomatic individuals (Chinello et al., 2015; Srinivas et al., 2001).

The use of multiple biomarkers has been suggested as possibly being the most promising way to improve diagnosis (Srinivas et al., 2001). Urinary biomarkers are extensively used, owing to its non-invasive diagnostic nature in many diseases including, prostate cancer, diabetic nephropathy, chronic kidney disease as well as for RCC (Chinello et al., 2015).

Mass spectrometry-based protein analysis of urine samples is a promising approach to obtain biomarker profiles for early detection. One of the main benefits of MALDI profiling is that signals are not required to be known to be used as biomarkers. On the other hand, the inability to identify such signals stays an obstacle towards its applicability and use as a routine laboratory test (Chinello et al., 2015).

 The study was conducted on eighty five subjects .They were divided into three groups; RCC (40 patients), benign renal neoplasms (15 patients) and control group (30 subjects). The study aimed to evaluate the urinary proteomic profile in RCC patients. Magnetic beads, hydrophobic-interaction chromatography (MB-HIC C8) was used, to fractionate all samples for proteomic profiling by matrix-assisted laser desorption/ionization time-of-flight mass spectrometry (MALDI-TOF MS) analysis. 

In univariate analysis, our study showed 64, 65 and 60 peaks when comparing the benign versus the controls, RCC versus benign and RCC versus the controls respectively. As multivariate analysis could give higher discriminatory information more than univariate analysis, the multivariate classification models were used to build the differentiating profiles between the different groups in our study. The GA model showed the best sensitivity and specificity in the three trials. ClinProt data and GA model showed that the RCC could be differentiated from the control with sensitivity of 88.7% and specificity of 73.2% built on GA model. This model originated in the training phase using about 50% of the data, in validation test using the other about 50% of the studied subjects.

Our results showed 64 peaks that differentiated between the benign and the control group among which 5 peaks were integrated (with mass to charge ratio of 8:1,913.17, 17:3,418.8, 15:3,388.67, 4:1,525.1, 25:4,173.41), 12 were PWKW significant (PWKW p> .05) and 2 were integrated and significant 17:3,418.8 and 25:4,173.41. These include 45 upregulated and 19 peaks down regulated.

For the RCC versus the benign groups our results showed 65 peaks from which 5 were integrated (with mass to charge ratio of 7:1,682, 40:5,247.36, 27:4,309.24, 18:3,428.41, 24:4,101.63), 28 significant(PWKW p> .05), one 

Regarding the RCC group versus the control, the trial showed 60 peaks among which 5 were integrated (with mass to charge ratio of 21:4,173.37, 35:5,246.64, 12:3,408.97, 24:4,308.97, 2:1,682.04),from which 1 was integrated and significant (with mass to charge ratio of 12:3,408.97) (PWKW p> .05), 36 upregulated and 24 were downregulated, from which peak no 35 and 24 were up regulated on the other hand 21 and 12 and 2 were down regulated.

In accordance with the current study, (Chinello et al., 2014), studied the urinary proteome in the same patient groups, including normal healthy subjects, ccRCC, and non-ccRCC patients using the same methodology which requires fractionation procedure based on C8 functionalized magnetic beads in combination with MALDI-TOF analysis. The study identified 2 ions at m/z 1,670 and 2,216 in MALDI-LM spectra and were identified as fragments of the human glycoprotein uromodulin (UMOD/THP). A common peak of m/z in the same range 1670 identified by Chinello et al. and peak 1,682 in the current study. Another study conducted by (Yang et al., 2014) was carried using serum rather than urine but applying the same technique performed in the current study MB-MALDI-TOF-MS method. It could generate serum peptidome profiles of clear cell RCC (ccRCC), to identify potential biomarkers for diagnosis as well as prognosis of this malignancy. In patients with clear cell RCC the study identified three candidate peaks, which were upregulated patients and had a tendency to be downregulated as in healthy control values after surgery. They were identified as peptide regions of RNA-binding protein 6 (RBP6), zinc finger tubulin beta chain (TUBB), and protein 3 (ZFP3) with the m/z values of 1,466.98, 5,905.23 and1618.22 respectively. A common mass range observation of peak sizes identified in the current study 5,246.64 which was upregulated and 1,682.04 which was downregulated in RCC patients when compared to controls.

Chinello et al., (2015) also conducted another study later using the same protocol but with different fractionation technique, which was the nano liquid chromatography (nano LC) and different ionization- method ESI–MS/ MS. Similarly, he found that some peptides were higher or lower represented in urine of RCC patients compared to control subjects. The m/z ratio of peptides that were up regulated were 1,755.8, 2,660.8, 4,849.1, and 4,866.2, while the down regulated ones were 1912.1, 1,934.2, 3,723.8, 3,990.0, 4,409.6 , 4,626.9, 4,751.5 and 6,261.4. These m/z ratio of peptides were in similar range as detected in the current work ranging 1-10kD.

Many researchers detected the urinary proteomic profile in RCC, using SELDI (Rogers et al., 2003; Wu et al., 2008; Alves et al., 2013). Urinary peptidome profiling with high-throughput methods such as MALDI-TOF MS or SELDI-TOF MS is a promising tool in nephrology research. Both methods are of particular interest because they enable rapid analyses of many individual samples in large-scale clinical studies. Although, MALDI-TOF MS seems to be more sensitive than SELDI-TOF MS.

Urinary proteomic profile using other platforms but in various genitourinary cancers was also applied. It has been studied in ovarian, prostate and bladder (Bauc et al., 2004). Majewski et al., (2012) used the urinary peptide profiling for detection of bladder cancer but he too did not identify the true nature of such peptide peaks.

In conclusion, using MALDI TOF spectra in this study might be useful in differentiating between benign and malignant renal masses which could be of help when kidney biopsies become technically challenging and hazardous. Furthermore it could be used for detection of relapses and monitoring therapy. Therefore further studies are required to confirm such findings together with applying an additional technique to identify such peaks.
